# Spin Hamiltonians in Magnets: Theories and Computations

**DOI:** 10.3390/molecules26040803

**Published:** 2021-02-04

**Authors:** Xueyang Li, Hongyu Yu, Feng Lou, Junsheng Feng, Myung-Hwan Whangbo, Hongjun Xiang

**Affiliations:** 1 Key Laboratory of Computational Physical Sciences (Ministry of Education), State Key Laboratory of Surface Physics, Department of Physics, Fudan University, Shanghai 200433, China; 16110190006@fudan.edu.cn (X.L.); 20110190060@fudan.edu.cn (H.Y.); flou16@fudan.edu.cn (F.L.); fjs@hfnu.edu.cn (J.F.); 2 Shanghai Qi Zhi Institute, Shanghai 200232, China; 3 School of Physics and Materials Engineering, Hefei Normal University, Hefei 230601, China; 4 Department of Chemistry, North Carolina State University, Raleigh, NC 27695-8204, USA; whangbo@ncsu.edu

**Keywords:** spin Hamiltonian, magnetism, energy-mapping analysis, four-state method, Green’s function method

## Abstract

The effective spin Hamiltonian method has drawn considerable attention for its power to explain and predict magnetic properties in various intriguing materials. In this review, we summarize different types of interactions between spins (hereafter, spin interactions, for short) that may be used in effective spin Hamiltonians as well as the various methods of computing the interaction parameters. A detailed discussion about the merits and possible pitfalls of each technique of computing interaction parameters is provided.

## 1. Introduction

The utilization of magnetism can date back to ancient China when the compass was invented to guide directions. Since the relationship between magnetism and electricity was revealed by Oersted, Lorentz, Ampere, Faraday, Maxwell, and others, more applications of magnetism have been invented, which include dynamos (electric generators), electric motors, cyclotrons, mass spectrometers, voltage transformers, electromagnetic relays, picture tubes, and sensing elements. During the information revolution, magnetic materials were extensively employed for information storage. The storage density, efficiency, and stability were substantially improved by the discovery and applications of the giant magnetoresistance effect [1,2], tunnel magnetoresistance [3,4,5,6,7,8,9], spin-transfer torques [10,11,12,13], etc. Recently, more and more novel magnetic states such as spin glasses [14,15], spin ice [16,17], spin liquid [18,19,20,21,22], and skyrmions [23,24,25,26,27,28] were found, revealing both theoretical and practical significance. For example, hedgehogs and anti-hedgehogs can be seen as the sources (monopoles) and the sinks (antimonopoles) of the emergent magnetic fields of topological spin textures [29], while magnetic skyrmions have shown promise as ultradense information carriers and logic devices [24].

To explain or predict the properties of magnetic materials, many models and methods have been invented. In this review, we will mainly focus on the effective spin Hamiltonian method based on first-principles calculations, and its applications in solid-state systems. In Section 2, we will introduce the effective spin Hamiltonian method. Firstly, in Section 2.1, the origin and the computing methods of the atomic magnetic moments are presented. Then, from Section 2.2, Section 2.3, Section 2.4, Section 2.5 and Section 2.6, different types of spin interactions that may be included in the spin Hamiltonians are discussed. Section 3 will discuss and compare various methods of computing the interaction parameters used in the effective spin Hamiltonians. In Section 4, we will give a brief conclusion of this review.

## 2. Effective Spin Hamiltonian Models

Though accurate, first-principles calculations are somewhat like black boxes (that is to say, they provide the final total results, such as magnetic moments and the total energy, but do not give a clear understanding of the physical results without further analysis), and have difficulties in dealing with large-scale systems or finite temperature properties. In order to provide an explicit explanation for some physical properties and improve the efficiency of thermodynamic and kinetic simulations, the effective Hamiltonian method is often adopted. In the context of magnetic materials where only the spin degree of freedom is considered, it can also be called the effective spin Hamiltonian method. Typically, the effective spin Hamiltonian models need to be carefully constructed and include all the possibly important terms; then the parameters of the models need to be calculated based on either first-principles calculations (see Section 3) or experimental data (see Section 3.3). Given the effective spin Hamiltonian and the spin configurations, the total energy of a magnetic system can be easily computed. Therefore, it is often adopted in Monte Carlo simulations [30] (or quantum Monte Carlo simulations) for assessing the total energy of many different configurations so that the finite temperature properties of magnetic materials can be studied. If the effects of atom displacements are taken into account, the effective Hamiltonians can also be applied to the spin molecular dynamics simulations [31,32,33], which is beyond the scope of this review.

In this review, we mainly focus on the classical effective spin Hamiltonian method where atomic magnetic moments (or spin vectors) are treated as classical vectors. In many cases, these classical vectors are assumed to be rigid so that their magnitudes keep constant during rotations. This treatment significantly simplifies the effective Hamiltonian models, and it is usually a good approximation, especially when atomic magnetic moments are large enough.

In this part, we shall first introduce the origin of the atomic magnetic moments as well as the methods of computing atomic magnetic moments. Then different types of spin interactions will be discussed.

### 2.1. Atomic Magnetic Moments

The origin of atomic magnetic moments is explained by quantum mechanics. Suppose the quantized direction is the *z*-axis, an electron with quantum numbers ($n,\;l\;,m_{l},\;m_{s}$) leads to an orbital magnetic moment $\mu_{l}=\;-\mu_{B}l$ and a spin magnetic moment $\mu_{s}=\;-g_{e}\mu_{B}s$, with their *z* components $\mu_{lz}=\;-m_{l}\mu_{B}$ and $\mu_{sz}=\;-g_{e}m_{s}\mu_{B}$, where $\mu_{B}=\;\frac{\left|e\right|\hbar }{2m}$ is the Bohr magneton and $g_{e}\approx 2$ is the *g*-factor for a free electron. The energy of a magnetic moment μ in a magnetic field B (magnetic induction) along *z*-direction is $-\mu \cdot B\;=\;-\mu_{z}B$.

Considering the Russell-Saunders coupling (also referred to as L-S coupling), which applies to most multi-electronic atoms, the total orbital magnetic moment and the total spin magnetic moment are $\mu_{L}=\;-\mu_{B}L$ and $\mu_{S}=\;-g_{e}\mu_{B}S$, respectively, where $L\;=\;\underset{i}{\sum }l_{i}$ and $S\;=\;\underset{i}{\sum }s_{i}$ are the summation over electrons. The quantum numbers of each electron can usually be predicted by Hund’s rules. Owing to the spin–orbit interaction, L and S both precess around the constant vector J = L+S. The time-averaged effective total magnetic moment is $\mu \;=\;-g_{J}\mu_{B}J$, where

(1) $$g_{J}=\;1+\frac{J\left(J+1\right)+S\left(S+1\right)-L\left(L+1\right)}{2J\left(J+1\right)}$$

The atomic magnetic moments discussed above are based on the assumption that the atoms are isolated. Taking the influence of other atoms and external fields into account, the orbital interaction theory, the crystal field theory [34], or the ligand field theory [35,36] may be a better choice for theoretically predicting atomic magnetic moments. Notice that half-filled shells lead to a total L = 0, and that in solids and molecules, orbital moments of electrons are usually quenched, resulting in an effective L = 0 [37] (counterexamples may be found for 4*f* elements or for 3*d*^7^ configurations as in Co(II)). Therefore typically $\mu \;=\;\mu_{S}$, with its *z* component $\mu_{z}=\;-g_{e}S_{z}\mu_{B}$ ($S_{z}$ is restricted to discrete values: S, S−1,…,−S). Usually, a nonzero $\mu_{S}$ results from singly filled (localized) *d* or *f* orbitals, while *s* and *p* orbitals are typically either doubly filled or vacant so that they have no direct contribution to the atomic magnetic moments. Therefore, when referring to atomic magnetic moments, usually only the atoms in the *d*- and *f*-transition series need to be considered.

The atomic magnetic moments can also be predicted numerically employing first-principles calculations. Nevertheless, we should notice that traditional Kohn–Sham density functional theory (DFT) calculations [38,39] (based on single-electron approximation) are not reliable for predicting atomic magnetic moments, and hence require the consideration of strong correlation effect among electrons, especially when dealing with localized *d* or *f* orbitals. Based on the Hubbard model [40], such problem can often be remedied by introducing an intra-atomic interaction with effective on-site Coulomb and exchange parameters, *U* and *J* [41] (or only one parameter $U^{\mathrm{eff}}=\;U-J$ in Dudarev’s approach [42]). This approach is the DFT+U method [41,42,43,44], including LDA+U (LDA: Local density approximation), LSDA+U (LSDA: Local spin density approximation), GGA+U (GGA: Generalized gradient approximation), and so forth, where “+U” indicates the Hubbard “+U” correction. The parameters *U* and *J* can be estimated according to experience or semi-empirically by seeking agreement with experimental results of some specific properties, which is convenient but not very reliable. Considering how the values of the parameters *U* and *J* affect the prediction of atomic magnetic moments and other physical properties, we may need to compute these parameters more rigorously. A typical approach is constrained DFT calculations [43,45,46,47], where the local *d* or *f* charges are constrained to different values in several calculations, so that the parameters *U* and *J* can be obtained. Another approach based on constrained random-phase approximation (cRPA) [48,49,50] allows for considering the frequency (or energy) dependence of the parameters. More methods of computing *U* and *J* are summarized in Ref. [43]. There are more accurate approaches for dealing with strong correlated systems like DFT + Dynamical Mean Field Theory (DFT+DMFT) [51,52,53,54,55] and Reduced Density Matrix Functional Theory (RDMFT) [56,57], but they are much more sophisticated and computationally demanding so that they may be impractical for large-scale calculations. Wave function (WF) methods, such as Complete Active Space Self-Consistent Field (CASSCF) [58,59,60,61], Complete Active Space second-order Perturbation Theory (CASPT2) [62,63,64], Complete Active Space third-order Perturbation Theory (CASPT3) [65], and Difference Dedicated Configuration Interaction (DDCI) [66,67,68], are also widely adopted by theoretical chemists for studying magnetic properties of materials (especially molecules), including atomic magnetic moments and magnetic interactions. These WF methods are also more accurate but more computationally demanding than the DFT+U method, more detailed discussions of which can be found in Ref. [69].

### 2.2. Heisenberg Model

The simplest effective spin Hamiltonian model is the classical Heisenberg model, which can be reduced to Ising model or XY model. The classical Heisenberg model can be written as

(2) $$H_{s\mathrm{pin}}\;=\;\underset{i,j>i}{\sum }J_{ij}S_{i}\cdot S_{j},$$

where $S_{i}$ and $S_{j}$ indicate the total spin vectors on atoms i and j, and the summation is over all relevant pairs (ij). Its form was suggested by Heisenberg, Dirac, and Van Vleck. Such an interaction comes from the energy splitting between quantized parallel (ferromagnetic, FM; triplet state) and antiparallel (antiferromagnetic, AFM; singlet state) spin configurations. $J_{ij}>0$ and $J_{ij}<0$ prefer AFM and FM configurations, respectively. There may be a difference in the factor such as −1 and $\frac{1}{2}$ between different definitions of $H_{s\mathrm{pin}}$, which is also the case in other models as will be discussed.

The spatial wave function of two electrons should possess the form of $\psi_{\pm }\;=\;\frac{1}{\sqrt{2}}\left[\psi_{a}\left(r_{1}\right)\psi_{b}\left(r_{2}\right)\pm \psi_{b}\left(r_{1}\right)\psi_{a}\left(r_{2}\right)\right]$, where $\psi_{a}$ and $\psi_{b}$ are any single-electron spatial wave functions. A parallel triplet spin state and an antiparallel singlet spin state should correspond to an antisymmetric ($\psi_{-}$) and a symmetric ($\psi_{+}$) spatial wave function, respectively. For given $\psi_{a}$ and $\psi_{b}$, the expectation value for the total energy of $\psi_{-}$ can be different from that of $\psi_{+}$, which gives a preference to the AFM or FM spin configuration. Whether the AFM or FM spin configuration is preferred depends on the circumstances, and their energy difference can be described as a Heisenberg term $J_{12}S_{1}\cdot S_{2}$.

In the simple case of an H_2_ molecule, an AFM singlet spin state is preferred, whose symmetric spatial wave function $\psi_{+}$ corresponds to a bonding state [70,71]. However, this leads to the total magnetic moment of zero because the two antiparallel electrons share the same spatial state. In another simple case, where $\psi_{a}$ and $\psi_{b}$ stands for two degenerate and orthogonal orbitals of the same atom, an FM triplet spin state is preferred, which is in agreement with Hund’s rules. Consider a set of orthogonal Wannier functions with $\phi_{n\lambda }\left(r\;-r_{\alpha }\right)$ resembling the *n*th atomic orbital with spin λ centered at the αth lattice site, and suppose there are *Nh* electrons each localized on one of the *N* lattice sites, each ion possessing *h* unpaired electrons. If these *h* electrons have the same exchange integrals with all the other electrons, the interaction resulting from the antisymmetrization of the wave functions can be expressed as

(3) $$H_{\mathrm{ex}}\;=\;\underset{\begin{array}{c} \alpha \alpha^{\prime }\\ nn^{\prime } \end{array}}{\sum }J_{nn^{\prime }}\left(r_{\alpha },r_{\alpha^{\prime }}\right)\left[\frac{1}{4}+S\left(r_{\alpha }\right)\cdot S\left(r_{\alpha^{\prime }}\right)\right]$$

which is called the Heisenberg exchange interaction [72]. After removing the constant terms, we can see such an interaction has the form of $H_{s\mathrm{pin}}\;=\;\underset{i,j>i}{\sum }J_{ij}S_{i}\cdot S_{j}$. 

Based on molecular orbital analysis using $\phi_{a}$ and $\phi_{b}$ to denote the singly filled *d* orbitals of the two spin-$\frac{1}{2}$ magnetic ions (i.e., *d*^9^ ions), Hay et al. [73] showed that the exchange interaction between the two ions can be approximately expressed as

(4) $$J\;=\;-2K_{ab}+\frac{{∆}^{2}}{U^{\mathrm{eff}}}\;=\;J_{F}+J_{\mathrm{AF}}\;$$

where

(5) $$K_{ab}\propto \langle \phi_{a}\left(1\right)\phi_{b}\left(2\right)|\frac{1}{r_{12}}|\phi_{b}\left(1\right)\phi_{a}\left(2\right)\rangle \;=\;\int \phi_{a}^{*}\left(r_{1}\right)\phi_{b}^{*}\left(r_{2}\right)\frac{1}{r_{12}}\phi_{b}\left(r_{1}\right)\phi_{a}\left(r_{2}\right)>0$$

(6) $$U^{\mathrm{eff}}\;=J_{aa}-J_{ab}\propto \langle \phi_{a}\left(1\right)\phi_{a}\left(2\right)|\frac{1}{r_{12}}|\phi_{a}\left(1\right)\phi_{a}\left(2\right)\rangle -\langle \phi_{a}\left(1\right)\phi_{b}\left(2\right)|\frac{1}{r_{12}}|\phi_{a}\left(1\right)\phi_{b}\left(2\right)\rangle >0$$

and ∆ indicates the energy gap between the bonding state and antibonding state constructed by $\phi_{a}$ and $\phi_{b}$. The two components of *J* have opposite signs, i.e., $J_{F}\;=\;-2K_{ab}<0$ and $J_{\mathrm{AF}}\;=\;\frac{{∆}^{2}}{U^{\mathrm{eff}}}>0$, which give preference to FM and AFM spin configurations, respectively. For general *d^n^* cases, more orbitals should be considered. Therefore the expression of J will be more complicated, but the exchange interaction can still be similarly decomposed into FM and AFM contributions [73]. An application of this analysis is that when calculating exchange parameter J using Dudarev’s approach of DFT+U with a parameter $U^{\mathrm{eff}}$, the calculated value of J should vary with $U^{\mathrm{eff}}$ approximately as $J\;=\;J_{F}+\frac{{∆}^{2}}{U^{\mathrm{eff}}}$ with $J_{F}$ and ${∆}^{2}$ to be fitted [74]. However, this is no longer correct when $U^{\mathrm{eff}}\to 0$.

Another mechanism that leads to FM spin configurations is the double exchange, in which the interaction between two magnetic ions is induced by spin coupling to mobile electrons that travels from one ion to another. A mobile electron has lower energy if the localized spins are aligned. Such a mechanism is essential in metallic systems containing ions with variable charge states [75,76].

**Figure 1:**
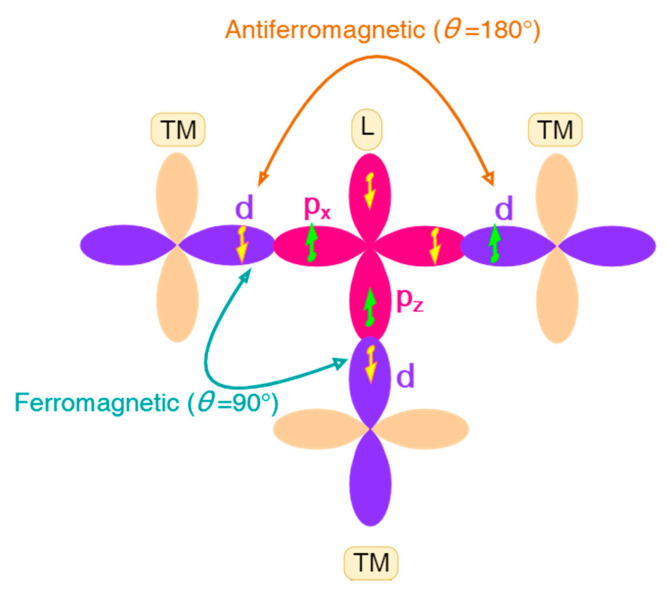
A schematic diagram of superexchange interactions between transition-metal (TM) ions both with more-than-half-filled *d*-shell. According to Goodenough–Kanamori (GK) rules, the 180° and the 90° cases favor antiferromagnetic (AFM) and ferromagnetic (FM) arrangements of TM ions, respectively. The main difference is whether the two electrons of L occupy the same *p* orbital, leading to different tendencies for the alignments of the two electrons of L that interact with two TM ions.

The superexchange is another important indirect exchange mechanism, where the interaction between two transition-metal (TM) ions is induced by spin coupling to two electrons on a non-magnetic ligand (L) ion that connects them, forming an exchange path of TM-L-TM type. Different mechanisms were proposed to explain the superexchange interaction. In Anderson’s mechanism [77,78], the superexchange results from virtual processes in which an electron is transferred from the ligand to one of the neighboring magnetic ions, and then another electron on the ligand couples with the spin of the other magnetic ion through exchange interaction. In Goodenough’s mechanism [79,80], the concept of semicovalent bonds was invented, where only one electron given by the ligand predominates in a semicovalent bond. Because of the exchange forces between the electrons on the magnetic ion and the electron given by the ligand, the ligand electron with its spin parallel to the net spin of the magnetic ion will spend more time on the magnetic ion than that with an antiparallel spin if the *d* orbital of the magnetic ion is less than half-filled, and vice versa. The magnetic atom and the ligand are supposed to be connected by a semicovalent bond or a covalent bond when they are near, or by an ionic bond (or possibly a metallic-like bond) otherwise. The superexchange interaction with semicovalent bonds existing is also called semicovalent exchange interaction. Kanamori summarized the dependence of the sign of the superexchange parameter (whether FM or AFM) on bond angle, bond type and number of *d* electrons (in different mechanisms), which is often referred as Goodenough–Kanamori (GK) rules [80,81,82]. For the 180° (bond angle) case, generally, AFM interaction is expected between cations of the same kind (counterexamples may exist for *d*^4^ cases such as Mn^3+^-Mn^3+^, where the sign depends on the direction of the line of superexchange), and FM interaction is expected between two cations with more-than-half-filled and less-than-half-filled *d*-shells, respectively [81]. For the 90° case, the results are usually the opposite [81]. A schematic diagram of superexchange interactions (between cations both with more-than-half-filled *d*-shell) is given in Figure 1. More details of the discussions can be found in Ref. [81] and Ref. [82]. 

A counterexample of the GK rules can be found in the layered magnetic topological insulator MnBi_2_Te_4_, which possesses intrinsic ferromagnetism [83]. In contrast, the prediction of the GK rules leads to a weak AFM exchange interaction between Mn ions. In Ref. [84], the presence of Bi^3+^ was found to be essential for explaining this anomaly: *d*^5^ ions in TM-L-TM spin-exchange paths would prefer FM coupling if the empty *p* orbitals of a nonmagnetic cation M (which is Bi^3+^ ion in the case of MnBi_2_Te_4_) hybridize strongly with those of the ligand L (but AFM coupling otherwise). Oleś et al. [85] pointed out that the GK rules may not be obeyed in transition metal compounds with orbital degrees of freedom (e.g., *d*^1^ and *d*^2^ electronic configurations) due to spin-orbital entanglement.

Exchange interactions between two TM ions also take place through the exchange paths of TM-L…L-TM type [86], referred to as super-superexchanges, where TM ions do not share a common ligand. Each TM ion of a solid forms a TML_n_ polyhedron (typically, n = 3–6) with the surrounding ligands L, and the unpaired spins of the TM ion are accommodated in the singly filled d-states of TML_n_. Since each *d*-state has a *d*-orbital of TM combined out-of-phase with the *p*-orbitals of L, the unpaired spin of TM does not reside solely on the *d*-orbital of TM, as assumed by Goodenough and Kanamori, but is delocalized into the *p*-orbitals of the surrounding ligands L. Thus, TM-L…L-TM type exchanges occur and can be strongly AFM when their L…L contact distances are in the vicinity of the van der Waals distance so that the ligand p-orbitals overlap well across the L…L contact. 

Another mechanism is the indirect coupling of magnetic moments by conduction electrons, referred to as Ruderman–Kittel–Kasuya–Yosida (RKKY) interaction [87,88,89,90]. This kind of interaction between two spins $S_{1}$ and $S_{2}$ is also proportional to $S_{1}\cdot S_{2}$ with an expression

(7) $$H_{\mathrm{RKKY}}\propto \underset{q}{\sum }\chi \left(q\right)e^{iq\cdot r_{21}}S_{1}\cdot S_{2}.$$

The magnetic dipole–dipole interaction (between magnetic moments $\mu_{1}$ and $\mu_{2}$ located at different atoms) with energy

(8) $$V\;=\;\frac{1}{R^{3}}\left[\mu_{1}\cdot \mu_{2}-3\left(\hat{R}\cdot \mu_{1}\right)\left(\hat{R}\cdot \mu_{2}\right)\right]$$

also has contributions to the bilinear term, but it is typically much weaker than the exchange interactions in most solid-state materials such as iron and cobalt. The characteristic temperature of dipole–dipole interaction (or termed as “dipolar interaction”) in magnetic materials is typically of the order of 1 K, above which no long-range order can be stabilized by such an interaction [37]. However, in some cases, such as in several single-molecule magnets (SMMs), the exchange interactions can be so weak that they are comparable to or weaker than dipolar interactions, thus the dipolar interactions must not be neglected [91].

For most magnetic materials, the Heisenberg interaction is the most predominant spin interaction. As a result, the simple classical Heisenberg model is able to explain the magnetic properties such as the ground states of spin configurations (FM or AFM) and the transition temperatures (Curie temperature for FM states or Néel temperature for AFM states) for many magnetic materials.

If some pairs of spins favor FM spin configurations while other pairs favor AFM configurations, frustration may occur, leading to more complicated and more interesting noncollinear spin configurations. For example, the FM effects of double exchange resulting from mobile electrons in some antiferromagnetic lattices give rise to a distortion of the ground-state spin arrangement and lead to a canted spin configuration [92]. A magnetic solid with moderate spin frustration lowers its energy by adopting a noncollinear superstructure (e.g., a cycloid or a helix) in which the moments of the ions are identical in magnitude but differ in orientation or a collinear magnetic superstructure (e.g., a spin density wave, SDW) in which the moments of the ions differ in magnitude but identical in orientation [93,94]. For a cycloid formed in a chain of magnetic ions, each successive spin rotates in one direction by a certain angle, so there are two opposite ways of rotating the successive spins hence producing two cycloids opposite in chirality but identical in energy. When these two cycloids occur with equal probability below a certain temperature, their superposition leads to a SDW [93,94]. On lowering the temperature further, the electronic structure of the spin-lattice relaxes to energetically favor one of the two chiral cycloids so that one can observe a cycloid state. The latter, being chiral, has no inversion symmetry and gives rise to ferroelectricity [95]. The spin frustration is also a potential driving force for topological states like skyrmions and hedgehogs [29].

### 2.3. The J Matrices and Single-Ion Anisotropy

The classical Heisenberg model can be generalized to a matrix form to include all the possible second-order interactions between two spins (or one spin itself):

(9) $$H_{s\mathrm{pin}}\;=\;\underset{i,j>i}{\sum }S_{i}^{T}J_{ij}S_{j}+\underset{i}{\sum }S_{i}^{T}A_{i}S_{i}\;$$

where $J_{ij}$ and $A_{i}$ are 3 × 3 matrices called the J matrix and single-ion anisotropy (SIA) matrix. The $J_{ij}$ matrix can be decomposed into three parts: The isotropic Heisenberg exchange parameter $J_{ij}\;=\;\left(J_{ij,xx}+J_{ij,yy}+J_{ij,zz}\right)/3$ as in the classical Heisenberg model, the antisymmetric Dzyaloshinskii–Moriya interaction (DMI) matrix $D_{ij}\;=\;\left(J_{ij}-J_{ij}^{T}\right)/2$ [96,97,98], and the symmetric (anisotropic) Kitaev-type exchange coupling matrix $K_{ij}\;=\;\left(J_{ij}+J_{ij}^{T}\right)/2-J_{ij}I$ (where I denotes a 3 × 3 identity matrix). Thus $J_{ij}\;=$ $J_{ij}I+D_{ij}+K_{ij}$ [99,100].

Now we analyze the possible origin of these terms by means of symmetry analysis. When considering interaction potential between (or among) spins, we should notice that the total interaction energy should be invariant under time inversion ($\left\{S_{k}\right\}\to \left\{-S_{k}\right\}$). Therefore, any odd order term in the spin Hamiltonian should be zero unless an external magnetic field is present when a term $-\underset{i}{\sum }\mu_{i}\cdot B=\;\underset{i}{\sum }g_{e}\mu_{B}B\cdot S_{i}$ should be added to the effective spin Hamiltonian. Ignoring the external magnetic field, the spin Hamiltonian should only contain even order terms, with the lowest order of significance being the second-order (the zeroth-order term is a constant and therefore not necessary). If the spin-orbit coupling (SOC) is negligible, the total effective spin Hamiltonian $H_{s\mathrm{pin}}$ should be invariant under any global spin rotations, therefore $H_{s\mathrm{pin}}$ should be expressed by only inner product terms of spins like terms proportional to $S_{i}\cdot S_{j}$, $\left(S_{i}\cdot S_{j}\right)\left(S_{k}\cdot S_{l}\right)$ and so on. That is to say, when SOC is negligible, the second-order terms in the $H_{s\mathrm{pin}}$ should only include the classical Heisenberg term $\underset{i,j>i}{\sum }J_{ij}S_{i}\cdot S_{j}$, which implies that those interactions described by $A_{i}$, $D_{ij}$, and $K_{ij}$ matrices all originate from SOC ($H_{\mathrm{SO}}=\lambda \hat{S}\cdot \hat{L}$). What is more, if the spatial inversion symmetry is satisfied by the lattice, $J_{ij}$ should be equal to $J_{ij}^{T}$ so that there will be no DMI ($D_{ij}=0$). That is to say, the DMI can only exist where the spatial inversion symmetry is broken.

The SIA matrix $A_{i}$ has only six independent components and is usually assumed to be symmetric. If we suppose the magnitude of the classical spin vector $S_{i}$ to be independent of its direction, the isotropic part $\frac{1}{3}\left(A_{i,xx}+A_{i,yy}+A_{i,zz}\right)I$ would be of no significance, and therefore $A_{i}$ would have only five independent components after subtracting the isotropic part from itself. It is evident that the $S_{i}^{T}A_{i}S_{i}$ prefers the direction of $S_{i}$ along the eigenvector of $A_{i}$ with the lowest eigenvalue. If this lowest eigenvalue is two-fold degenerate, the directions of $S_{i}$ favored by SIA will be those belonging to the plane spanned by the two eigenvectors that share the lowest eigenvalue, in which case we say the ion *i* has easy-plane anisotropy. On the contrary, if the lowest eigenvalue is not degenerate while the higher eigenvalue is two-fold degenerate, we say the ion *i* has eas*y*-axis anisotropy. In these two cases (easy-plane or eas*y*-axis anisotropy), by defining the direction of *z*-axis parallel to the nondegenerate eigenvector, the $S_{i}^{T}A_{i}S_{i}$ part would be simplified to $A_{i,zz}{\left(S_{i}^{z}\right)}^{2}$ with only one independent component. The eas*y*-axis anisotropy has been found to be helpful in stabilizing the long-range magnetic order and enhancing the Curie temperature in two-dimensional or quasi-two-dimensional systems [101]. The easy-plane anisotropy in three-dimensional ferromagnets can lead to the effect called “quantum spin reduction”, where the mean spin at zero temperature has a value lower than the maximal one due to the quantum fluctuations [101,102]. Recently, several materials with unusually large easy-plane or eas*y*-axis anisotropy were found [103,104,105], which, as single-ion magnets (SIMs), are promising for applications such as high-density information storage, spintronics, and quantum computing.

The DMI matrix $D_{ij}$ is antisymmetric and therefore has only three independent components, which can be expressed by a vector $D_{ij}$ with $D_{ij,x}=D_{ij,yz}$, $D_{ij,y}=D_{ij,zx}$, and $D_{ij,z}=D_{ij,xy}$. Thus, the DMI can be expressed by cross product: $S_{i}^{T}D_{ij}S_{j}=D_{ij}\cdot \left(S_{i}\times S_{j}\right)$. Such an interaction prefers the vectors $S_{i}$ and $S_{j}$ to be orthogonal to each other, with a rotation (of $S_{j}$ relative to $S_{i}$) around the direction of $-D_{ij}$. Together with Heisenberg term $J_{ij}S_{i}\cdot S_{j}$, the preferred rotation angle between $S_{i}$ and $S_{j}$ relative to the collinear state preferred by the Heisenberg term would be $\arctan\frac{\left|D_{ij}\right|}{\left|J_{ij}\right|}$. In Ref. [106], the DMI is shown to determine the chirality of the magnetic ground state of Cr trimers on Au(111). The DMI is also important in explaining the skyrmion states in many materials such as MnSi and FeGe [24,26,27,29,107,108,109,110]. Materials with skyrmion states induced by DMI usually have a large ratio of $\frac{\left|D_{1}\right|}{\left|J_{1}\right|}$ (typically 0.1~0.2) where the subscript “1” means nearest pairs [24,100]. In Ref. [100], strong enough DMI for the existence of helical cycloid phases and skyrmionic states are predicted in Cr(I,X)_3_ (X = Br or Cl) Janus monolayers (e.g., for Cr(I,Br)_3_, supposing $\left|S_{i}\right|=\frac{3}{2}$ for any *i*, the corresponding interaction parameters are computed as $J_{1}=-1.800$ meV and $\left|D_{1}\right|=0.270$ meV, thus $\frac{\left|D_{1}\right|}{\left|J_{1}\right|}=0.150$), though monolayers such as CrI_3_ only exhibit an FM state for lack of DMI. In Ref. [110], the nonreciprocal magnon spectrum (and the associated spectral weights) of MnSi, as well as its evolution as a function of magnetic field, is explained by a model including symmetric exchange, DMI, dipolar interactions, and Zeeman energy (related to the magnetic field).

The Kitaev matrix $K_{ij}$ has five independent components as a symmetric matrix with zero trace. For the specific cases when $S_{i}$ and $S_{j}$ are parallel to each other (pointing in the same direction), $S_{i}^{T}K_{ij}S_{j}$ would perform like $S_{i}^{T}A_{i}S_{i}$ and show preference to the direction with the lowest eigenvalue of $K_{ij}$; while when $S_{i}$ and $S_{j}$ are antiparallel to each other, $S_{i}^{T}K_{ij}S_{j}$ would prefer the direction with the highest eigenvalue of $K_{ij}$. The difference between the highest and lowest eigenvalue of $K_{ij}$ can be defined as $K_{ij}$ (a scalar), which characterizes the anisotropic contribution of $K_{ij}$. Generally, the favorite direction of the spins is decided by both SIA and Kitaev interactions. The long-range ferromagnetic order in monolayer CrI_3_ was explained by the anisotropic superexchange interaction since the Cr-I-Cr bond angle is close to 90° [111]. In Ref. [112], the interplay between the prominent Kitaev interaction and SIA was studied to explain the different magnetic behaviors of CrI_3_ and CrGeTe_3_ naturally. For CrI_3_, supposing $\left|S_{i}\right|=\frac{3}{2}$ for any *i*, the $J_{ij}$ and $K_{ij}$ parameters between nearest pairs are computed as −2.44 and 0.85 meV, respectively; while the only independent component $A_{i,zz}$ of $A_{i}$ is −0.26 meV. For CrGeTe_3_, these three parameters are calculated to be −6.64, 0.36, and 0.25 meV, respectively. These two kinds of interactions are induced by SOC of the heavy ligands (I or Te) in these two materials (rather than the commonly believed Cr ions). Among different types of quantum spin liquids (QSLs), the exactly solvable Kitaev model with a ground state being QSL (with Majorana excitations) [113] has attracted much attention. Materials that achieve the realization of such Kitaev QSLs as α-RuCl_3_ [114,115,116] and (Na_1-*x*_Li*_x_*)_2_IrO_3_ [117,118] (with an effective *S* = 1/2 spin value) with honeycomb lattices are discovered. A possible Kitaev QSL state is also predicted in epitaxially strained Cr-based monolayers with *S* = 3/2, e.g., CrSiTe_3_ and CrGeTe_3_ [119].

### 2.4. Fourth-Order Interactions without SOC

Sometimes, higher-order interactions are also crucial for explaining the magnetic properties of some materials, especially if the magnetic atoms have large magnetic moments or if the system is itinerant. As mentioned in Section 2.3, when SOC can be ignored, the effective spin Hamiltonian should only include inner product terms of spins. Besides second-order Heisenberg terms like $J_{ij}S_{i}\cdot S_{j}$, the terms with the next lowest order (which is fourth-order) are biquadratic (exchange) terms like $K_{ij}{\left(S_{i}\cdot S_{j}\right)}^{2}$, three-body fourth-order terms like $K_{ijk}\left(S_{i}\cdot S_{j}\right)\left(S_{i}\cdot S_{k}\right)$, and four-spin ring coupling terms like $K_{ijkl}\left(S_{i}\cdot S_{j}\right)\left(S_{k}\cdot S_{l}\right)$. That is to say, when SOC and external magnetic field are ignored, keeping the terms with orders no higher than fourth, the effective spin Hamiltonian can be expressed as

(10) $$H_{s\mathrm{pin}}=\underset{i,j>i}{\sum }J_{ij}S_{i}\cdot S_{j}+\underset{i,j>i}{\sum }K_{ij}{\left(S_{i}\cdot S_{j}\right)}^{2}+\underset{\begin{array}{c} i,j,\\ k>j \end{array}}{\sum }K_{ijk}\left(S_{i}\cdot S_{j}\right)\left(S_{i}\cdot S_{k}\right)+\underset{\begin{array}{c} i,j>i,\\ k>i,\\ l>k \end{array}}{\sum }K_{ijkl}\left(S_{i}\cdot S_{j}\right)\left(S_{k}\cdot S_{l}\right)$$

The biquadratic terms have been found to be important in many systems, such as MnO [120,121], YMnO_3_ [74], TbMnO_3_ [122], iron-based superconductor KFe_1.5_Se_2_ [123], and 2D magnets [124]. In the case of TbMnO_3_, besides the biquadratic terms, the four-spin couplings are also found to be important in explaining the non-Heisenberg behaviors [122]; the three-body fourth-order terms are also found to be important in simulating the total energies of different spin configurations [125] (a list of the fitted values of each important interaction parameter in TbMnO_3_ is provided in the supplementary material of Ref. [125]). According to Ref. [126], in a Heisenberg chain system constructed from alternating $S>\frac{1}{2}$ and $S=\frac{1}{2}$ site spins, the additional isotropic three-body fourth-order terms are found to stabilize a variety of partially polarized states and two specific non-magnetic states including a critical spin-liquid phase and a critical nematic-like phase. In Ref. [127], the four-spin couplings were found to have a large effect on the energy barrier preventing skyrmions (or antiskyrmions) collapse into the ferromagnetic state in several transition-metal interfaces.

### 2.5. Chiral Magnetic Interactions Beyond DMI

**Figure 2:**
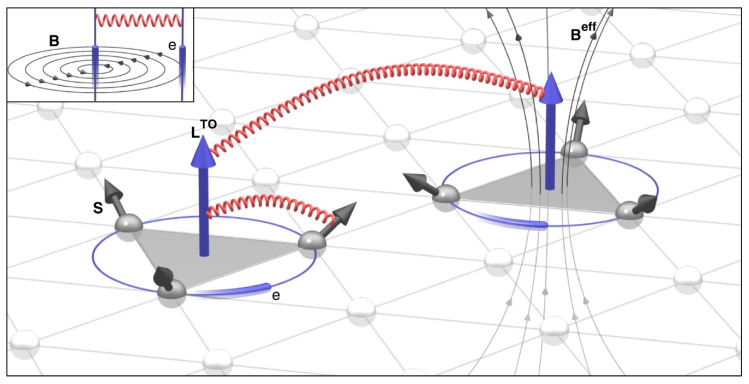
Schematic diagrams of chiral–chiral interactions (CCI) and spin–chiral interactions (SCI), as provided by S. Grytsiuk et al. in Ref. [128]. Spins and topological orbital moments (TOMs) are denoted as black arrows and blue arrows, respectively. CCI can be regarded as interactions between TOMs, while SCI can be interpreted as interactions between TOM and local spins.

Some high-order terms containing cross products of spins may also be necessary for fitting the models to the total energy or explaining some certain magnetic properties. Due to the chiral properties of these interactions like DMI, it is possible that they can also lead to, or explain, intriguing noncollinear spin textures such as skyrmions. In Ref. [128], topological–chiral interactions are found to be very prominent in MnGe, which includes chiral–chiral interactions (CCI) with the form

(11) $$\kappa_{ijki^{\prime }j^{\prime }k^{\prime }}^{\mathrm{CC}}\left[S_{i}\cdot \left(S_{j}\times S_{k}\right)\right]\left[S_{i^{\prime }}\cdot \left(S_{j^{\prime }}\times S_{k^{\prime }}\right)\right]$$

whose local part has the form

(12) $$\kappa_{ijk}^{\mathrm{CC}}{\left[S_{i}\cdot \left(S_{j}\times S_{k}\right)\right]}^{2}$$

and spin–chiral interactions (SCI) with the form

(13) $$\kappa_{ijk}^{\mathrm{SC}}\left(\tau_{ijk}\cdot S_{i}\right)\left[S_{i}\cdot \left(S_{j}\times S_{k}\right)\right]$$

where the unit vector $\tau_{ijk}\propto \left(R_{j}-R_{i}\right)\times \left(R_{k}-R_{i}\right)$ is the surface normal of the oriented triangle spanned by the lattice sites $R_{i}$, $R_{j}$ and $R_{k}$. The local scalar spin chirality $\chi_{ijk}=S_{i}\cdot \left(S_{j}\times S_{k}\right)$ among triplets of spins can be interpreted as a fictitious effective magnetic field $B^{e\mathrm{ff}}\propto \chi_{ijk}\tau_{ijk}$, which leads to topological orbital moments $L^{TO}$ (TOM) [129,130,131,132,133,134] arising from the orbital current of electrons hopping around the triangles. The TOM is defined as

(14) $$L_{i}^{TO}=\underset{\left(jk\right)}{\sum }L_{ijk}^{TO}=\underset{\left(jk\right)}{\sum }\kappa_{ijk}^{TO}\chi_{ijk}\tau_{ijk}$$

where $\kappa_{ijk}^{TO}$ is the local topological orbital susceptibility. CCI corresponds to the interaction between pairs of topological orbital currents (or TOMs), whose local part can be interpreted as the orbital Zeeman interaction $L_{i}^{TO}\cdot B^{\mathrm{eff}}$. SCI arises from the SOC, which couples the TOM to single spin magnetic moments. An illustration of CCI and SCI is provided in Figure 2. Considerations of CCI and SCI improved the fitting of the total energy in MnGe (see details in Ref. [128]). Moreover, the authors showed the possibility that the CCI may lead to three-dimensional topological spin states and therefore may be vital in deciding the ground state of the spin configurations of MnGe, which was found to be a three-dimensional topological lattice (possibly built up with hedgehogs and anti-hedgehogs) experimentally [135].

A new type of chiral pair interaction $C_{ij}\cdot \left(S_{i}\times S_{j}\right)\;\left(S_{i}\cdot S_{j}\right)$, named as chiral biquadratic interaction (CBI), which is the biquadratic equivalent of the DMI, was derived from a microscopic model and demonstrated to be comparable in magnitude to the DMI in magnetic dimers made of 3*d* elements on Pt(111), Pt(001), Ir(111) and Re(0001) surface with strong SOC [136]. Similar but generalized chiral interactions such as $D_{ijjk}\cdot \left(S_{j}\times S_{k}\right)\;\left(S_{i}\cdot S_{j}\right)$ and $D_{ijkl}\cdot \left(S_{k}\times S_{l}\right)\;\left(S_{i}\cdot S_{j}\right)$, named four-spin chiral interactions, were discussed in Ref. [137], and they are found to be important in predicting a correct chirality for a spin spiral state of Fe chains deposited on the Re(0001) surface.

When there is a magnetic field, for a nonbipartite lattice, the magnetic field can couple with the spin and produce a new term of the form $J_{ijk}S_{i}\cdot \left(S_{j}\times S_{k}\right)=J_{ijk}\chi_{ijk}$ [138,139], which can be termed the three-spin chiral interaction (TCI) [140]. Such a chiral term can induce a gapless line in frustrated spin-gapped phases, and a critical chiral strength can change the ground state from spiral to Néel quasi-long-range-order phase [138]. This chiral term is also found to produce a chiral spin liquid state [141], where the time-reversal symmetry is broken spontaneously by the emergence of long-range order of scalar chirality [142]. 

### 2.6. Expansions of Magnetic Interactions

In general, a complete basis can be used to expand the spin interactions. One example is the spin-cluster expansion (SCE) [143,144,145], where unit vectors denoting the directions of spins are used as independent variables and spherical harmonic functions are used in the expressions of the basis functions. When SOC and the external magnetic field are not important, as mentioned in Section 2.3, only inner products of spins need to be considered. Consequently, the expansion can be

(15) $$H_{\mathrm{spin}}=E_{0}+\overset{\infty }{\underset{n=1}{\sum }}J_{n}\left(\underset{n\mathrm{th}\;\mathrm{nearest}\;\mathrm{pairs}\;\langle k,l\rangle }{\sum }e_{k}\cdot e_{l}\right)+\overset{\infty }{\underset{n^{\prime }=1}{\sum }}J_{n^{\prime }}^{\prime }\underset{n^{\prime }\mathrm{th}\;\mathrm{type}\;}{\sum }\left(e_{k}\cdot e_{l}\right)\left(e_{m}\cdot e_{n}\right)+\cdots$$

as used in Ref. [125]. When using expansions of spin vectors (or directions of the spins), suitable truncations on interaction distances and interaction orders are needed. Otherwise, the number of terms would be infinite, and as a result, the problem would be unsolvable.

## 3. Methods of Computing the Parameters of Effective Spin Hamiltonian Models

In this part, we mainly discuss the methods of computing the spin interaction parameters based on first-principles calculations of crystals, where periodic boundary conditions are tacitly supposed. These methods include different kinds of energy-mapping analysis (see Section 3.1) and Green’s function method based on magnetic-force linear response theory (see Section 3.2). Discussions on the rigid spin rotation approximation and other assumptions are provided in Section 3.3. The cases of clusters, where periodic boundary conditions do not exist, will be briefly discussed in Section 3.3. Methods of obtaining spin interaction parameters from experiments will also be briefly mentioned in Section 3.3.

### 3.1. Energy-Mapping Analysis

In an energy-mapping analysis, we do several first-principles calculations to assess the total energies of different spin configurations. Then, we use the effective spin Hamiltonian to provide the expressions of the total energies of these spin configurations (with the expressions containing several undetermined parameters). By mapping the total energy expressions given by the effective spin Hamiltonian model to the results of first-principles calculations, the values of the undetermined parameters can be estimated. There are several types of energy-mapping analysis. For the first type, a minimal number of configurations are used, and a concrete expression for calculating the parameters can be given in advance. An example is by mapping between the eigenvalues and eigenfunctions of exact Hamiltonians and the effective spin Hamiltonian models (typically the Heisenberg model) to estimate the exchange parameters for relatively simple systems [146,147]. Several broken symmetry (BS) approaches are also of this type, where broken-symmetry states (instead of eigenstates of exact Hamiltonians) are adopted for energy mapping between the models and results of first-principles calculations [147,148]. A typical example of BS approach is the four-state method [148,149] where four special states are chosen for calculating each component of the parameters, which will be introduced in Section 3.1.1. For the second type, more configurations are used, and the parameters in the supposed effective spin Hamiltonian model are determined by employing least-squares fitting, which will be introduced in Section 3.1.2. The third type is similar to the second one, but the concrete form of the effective spin Hamiltonian model is not determined in advance. In the beginning, one includes many terms in the mode Hamiltonians. The relevance of each individual term depends on the fitting performance with respect to first-principles calculations. While selecting the relevant terms for a model Hamiltonian, it is important to search for the minimal Hamiltonian for a given magnetic system, namely, the one with the minimal number of parameters that capture its essential physics. This type of energy-mapping analysis will be introduced in Section 3.1.3.

In this section, we will mainly focus on the applications in solid state systems with periodic boundary conditions. The total energy of a designated configuration (which is usually a broken-symmetry state) is typically provided by first-principles calculations (e.g., DFT+U calculations) with constrained directions of magnetic moments.

#### 3.1.1. Four-State Method

The energy-mapping analysis based on four ordered spin states [148,149], also referred to as the four-state method, assumes the effective spin Hamiltonian include only second-order terms (i.e., isotropic Heisenberg terms, DMI terms, Kitaev terms, and SIA terms). Each component of the parameters like $J_{ij}$, $D_{ij,x}$, $A_{i,xy}$, ($A_{i,yy}-A_{i,xx}$) and ($A_{i,zz}-A_{i,xx}$) can be obtained by first-principles calculations for four specified spin states [148]. Taking the isotropic Heisenberg parameter $J_{ij}$ for example, with the spin-orbit coupling (SOC) switched off during the first-principles calculations, we use $E_{ij,\alpha \beta }$ (α,β=↑,↓) to denote the energy of the configuration where spin *i* is parallel or antiparallel to the *z* direction (if α=↑or ↓, respectively), spin *j* is parallel or antiparallel to the *z* direction (if β= ↑or↓, respectively), and all the spins except *i* and *j* are kept unchanged in the four states (which will be referred to as the “reference configuration”, usually chosen to be a low-energy collinear state). Then $J_{ij}$ can be expressed as

(16) $$J_{ij}=\frac{E_{ij,↑↑}+E_{ij,↓↓}-E_{ij,↑↓}-E_{ij,↓↑}}{4S^{2}}$$

**Figure 3:**
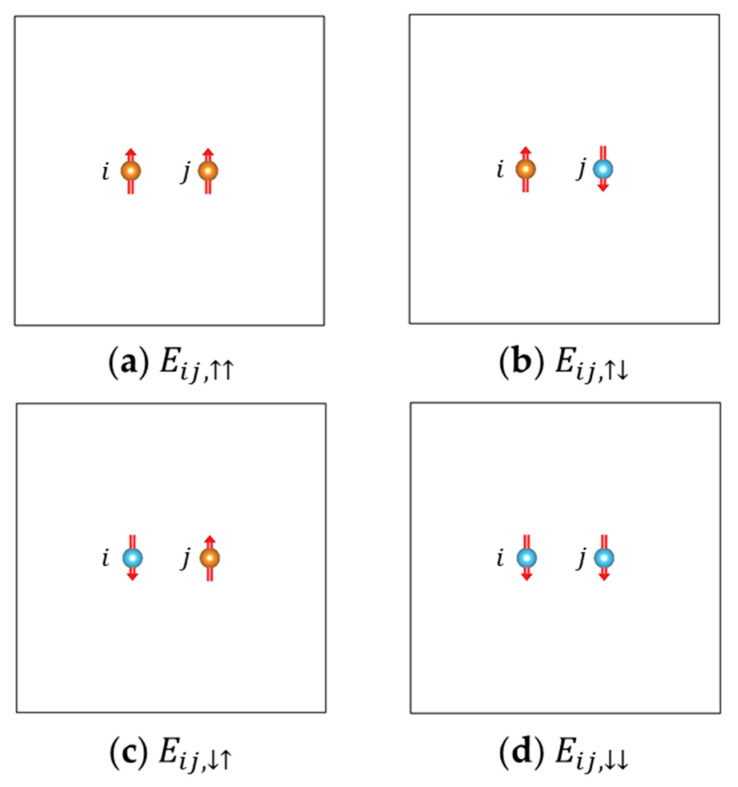
Schematic diagrams of the four states adopted in the four-state method for computing $J_{ij}$. Except atoms *i* and *j*, all the other atoms are omitted in this diagram (which are kept the same in the four states). Spins pointing up and down are indicated with orange and blue balls, respectively. The total energies given by first-principles calculations corresponding to these four states are denoted as (**a**) $E_{ij,↑↑}$, (**b**) $E_{ij,↑↓}$, (**c**) $E_{ij,↓↑}$, and (**d**) $E_{ij,↓↓}$, respectively.

The schematic diagrams of these four states are shown in Figure 3.

In general, the second-order effective spin Hamiltonian takes the form of

(17) $$H_{s\mathrm{pin}}=\underset{i,j>i}{\sum }S_{i}^{T}J_{ij}S_{j}+\underset{i}{\sum }S_{i}^{T}A_{i}S_{i}$$

and each component of the matrix $J_{ij}$ and $A_{i}$ can also be obtained by first-principles calculations for four specified spin states. To compute $J_{ij,ab}$ (a,b=x,y,z), we use $E_{ij,ab,\alpha \beta }$ (α,β= ↑,↓) to denote the energy of the configuration where spin *i* is parallel or antiparallel to the *a* direction (if α= ↑or↓, respectively), spin *j* is parallel or antiparallel to the *b* direction (if β= ↑or↓, respectively), and all the spins except for *i* and *j* are kept unchanged and parallel to the *c*-axis (c=x,y or z, c≠a, c≠b) with an appropriate reference configuration and kept unchanged. Then, $J_{ij,ab}$ can be expressed as

(18) $$J_{ij,ab}=\frac{E_{ij,ab,↑↑}+E_{ij,ab,↓↓}-E_{ij,ab,↑↓}-E_{ij,ab,↓↑}}{4S^{2}}$$

To compute $A_{i,ab}$ (a,b=x,y,z with a≠b), we use $E_{i,ab,\alpha \beta }$ (α,β= ↑,↓) to denote the energy of the configuration where spin *i* is parallel to the direction whose *a* component is $\pm \frac{\sqrt{2}}{2}$ (for α= ↑or↓, respectively), *b* component is $\pm \frac{\sqrt{2}}{2}$ (for β= ↑or↓, respectively), and the other component is 0. Here all the spins except for *i* and *j* are parallel to the *c*-axis (c=x,y or z, c≠a, c≠b) with an appropriate reference configuration. Then, $A_{i,ab}$ can be expressed as

(19) $$A_{i,ab}=\frac{E_{i,ab,↑↑}+E_{i,ab,↓↓}-E_{i,ab,↑↓}-E_{i,ab,↓↑}}{4S^{2}}$$

To compute $\left(A_{i,aa}-A_{i,bb}\right)$ (a,b=x,y,z with a≠b), we use $E_{i,ab,\alpha \beta }$ (α,β= ↑,↓or 0) to denote the energy of the configuration where spin *i* is parallel to the direction whose *a* component is ±1 or 0 (if α= ↑, ↓or 0, respectively), *b* component is ±1 or 0 (if β= ↑, ↓or 0, respectively) and the other component is 0, while all the spins except *i* are parallel to the *c*-axis (c=x,y or z, c≠a, c≠b) with an appropriate reference configuration. Then $\left(A_{i,aa}-A_{i,bb}\right)$ can be expressed as

(20) $$A_{i,aa}-A_{i,bb}=\frac{E_{i,ab,↑0}+E_{i,ab,↓0}-E_{i,ab,0↑}-E_{i,ab,0↓}}{4S^{2}}$$

It is easy to verify that, by employing this four-state method, each component of the $J_{ij}$ and $A_{i}$ can be obtained with the effects of other second-order terms entirely cancelled. Now we take the effects of fourth-order terms (without SOC) into account and check if the algorithms for computing $J_{ij}$ and $A_{i}$ are still rigorous. For $A_{i}$, we can find out that the effects of all these terms are correctly cancelled. For $J_{ij}$, the effects of biquadratic terms, three-body fourth-order terms, and most of the four-spin ring coupling terms are perfectly cancelled, while only the terms like $\left(S_{i}\cdot S_{j}\right)\left(S_{k}\cdot S_{l}\right)$ (k,l≠i,j) will interfere with the calculation of $J_{ij}$ because $\left(S_{k}\cdot S_{l}\right)$ is constant during the calculation and therefore mixed with the contribution of $J_{ij}\left(S_{i}\cdot S_{j}\right)$. The error of the calculated $J_{ij}$ originated from four-spin ring coupling terms

(21) $$\underset{\begin{array}{c} i,j>i,\\ k>i,\\ l>k \end{array}}{\sum }K_{ijkl}\left(S_{i}\cdot S_{j}\right)\left(S_{k}\cdot S_{l}\right)$$

is given by

(22) $$\underset{\begin{array}{c} k\neq i\;or\;j\\ l\neq i\;or\;j\\ \left(l>k\right) \end{array}}{\sum }K_{ijkl}\left(S_{k}\cdot S_{l}\right)$$

while there is no easy way to get rid of this problem perfectly. Other parts of the $J_{ij}$ (including $D_{ij}$ and $K_{ij}$) are not affected by these fourth-order terms (without SOC), but by other types of fourth-order terms (like four-spin chiral interactions) if SOC is taken into account (because of the similar reason).

**Figure 4:**
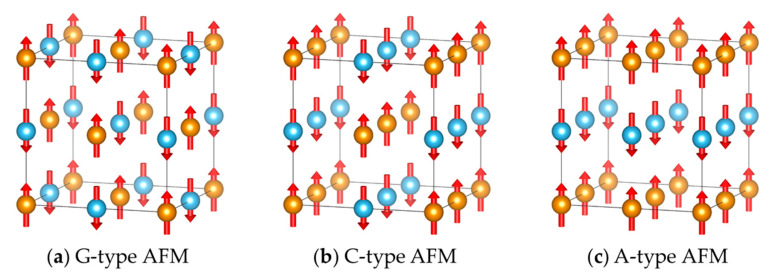
Schematic diagrams of (**a**) G-type AFM, (**b**) C-type AFM, and (**c**) C-type AFM states. Spins pointing to two opposite directions (e.g., up and down) are indicated with orange and blue balls, respectively.

In Ref. [122], the four-spin ring coupling interaction is found to be important in TbMnO_3_, and therefore leads to instability in calculating the Heisenberg parameters using the four-state method when changing the reference configurations. This problem is remedied by calculating the Heisenberg parameter $J_{ij}$ twice with the four-state method using the FM and A-type AFM (A-AFM, see Figure 4c) as the reference configurations, and use their average value as the final estimation of $J_{ij}$. The parameter of the vital ring coupling interaction is obtained by calculating the difference between the two calculated $J_{ij}$ values (with FM and A-AFM reference configurations, respectively). This effective remedy is based on the assumption that only one kind of ring coupling interaction is essential. However, if there are more kinds of significant ring coupling or if we do not know which ring coupling is essential in advance, such a method of calculating $J_{ij}$ is still not very trustworthy. Nevertheless, it is found that by taking the average of the calculated $J_{ij}$ with four-state method with FM and G-type AFM (G-AFM, see Figure 4a) reference configurations, the influences of $K_{ijkl}\left(S_{i}\cdot S_{j}\right)\left(S_{k}\cdot S_{l}\right)$ with *k* and *l* being nearest pairs are eliminated. The terms $K_{ijkl}\left(S_{i}\cdot S_{j}\right)\left(S_{k}\cdot S_{l}\right)$ with non-nearest pairs of *k* and *l* still interfere with the calculation of $J_{ij}$, but they are generally very weak. Therefore, such a remedy to calculate $J_{ij}$ using the four-state method should work well in most cases. In cases when a G-AFM state (in which all the nearest pairs of spins are antiparallelly aligned) cannot be defined (e.g., a triangular or a Kagomé lattice), there may be more than two reference configurations to use in the four-state method so as to eliminate the effects of $K_{ijkl}\left(S_{i}\cdot S_{j}\right)\left(S_{k}\cdot S_{l}\right)$ terms with non-nearest pairs of *k* and *l*. These reference configurations need to be designed carefully according to the specific circumstances to get rid of the effects of $K_{ijkl}\left(S_{i}\cdot S_{j}\right)\left(S_{k}\cdot S_{l}\right)$ terms as much as possible.

The main advantages of the four-state method are its relatively small amount of first-principles calculations and its relatively good cancellations of other relevant terms. A weakness is that it cannot analyze the uncertainties of the parameters, so that we do not know how precise those estimated values are. Another weakness, which is also shared with other energy-mapping analysis methods, is that the computed $J_{ij}$ between $S_{i}$ and $S_{j}$ is actually the sum over $J_{ij^{\prime }}$ with any lattice vector $R=r_{j^{\prime }}-r_{j}$. Therefore, to get rid of the effects of other spin pairs, a relatively large supercell is needed.

The four-state method can also be generalized to compute biquadratic parameters, where the SOC needs to be switched off during the first-principles calculations. For calculating $K_{ij}$ in the term $K_{ij}{\left(S_{i}\cdot S_{j}\right)}^{2}$, we can let $S_{i}$ pointing to the (1,0,0) direction, $S_{j}$ pointing to the (1,0,0), (−1,0,0), $\left(\frac{1}{\sqrt{2}},\frac{1}{\sqrt{2}},0\right)$ and $\left(-\frac{1}{\sqrt{2}},-\frac{1}{\sqrt{2}},0\right)$ directions, with other spins parallel to the *z*-axis. The corresponding total energies are denoted as $E_{1}$, $E_{2}$, $E_{3}$, and $E_{4}$, respectively. Then the $K_{ij}$ can be expressed as

(23) $$K_{ij}=E_{1}+E_{2}-E_{3}-E_{4}$$

It can be easily checked that the effects of other terms not higher than fourth order are totally eliminated. Therefore, this approach of calculating $K_{ij}$ should be relatively rigorous theoretically.

Note that the four-state methods [148,149] could also give the derivatives of exchange interactions with respect to the atomic displacements without doing additional first-principles calculations due to the Hellmann-Feynman theorem. These derivatives are useful for the study of spin-lattice coupling related phenomena.

#### 3.1.2. Direct Least Squares Fitting

Another type of energy-mapping analysis, instead of the four-state method, uses more first-principles calculations with different spin configurations and fits the results to the effective spin Hamiltonian using the least-squares method to estimate the parameters [74,122,123,124]. Ways of choosing spin configurations can depend on which parameters to estimate.

In Ref. [74], for the four Mn sites in a unit cell of YMnO_3_, the polar and azimuthal angles (θ,φ) of their spins are given by (0, 0), (0, 0), $\left(\theta ,\;\frac{3\pi }{2}\right)$, and $\left(\theta ,\;\frac{\pi }{2}\right)$, respectively. By changing the θ from 0° to 180°, different configurations are produced. If the effective spin Hamiltonian only contains Heisenberg terms, there will be a systematic deviation between the predicted value given by the effective Hamiltonian and the calculated value given by first-principles calculations. Such a deviation is well remedied by adding biquadratic exchange interactions into the effective Hamiltonian model. Thus, the biquadratic parameters can be fitted. Similar approaches were adopted by others to calculate and show the importance of biquadratic parameters and topological chiral–chiral contributions [122,123,124,128]. Apart from using an angular variable for generating spin configurations, using two or more variables is also practicable, or randomly chosen directions [106] can also be considered. Thus, more diverse configurations will be produced. The least-squares fitting will also work, but whether a systematic deviation exists will not be as apparent as the case when only one angular variable is used for generating configurations, and the fitting task may be more laborious. 

The main virtues of this method are that the reliability of the model can be checked by the fitting performance and that the uncertainties of the parameters can be estimated if needed. This method is especially suitable for calculations of biquadratic parameters and topological CCI. However, when talking about calculations of Heisenberg parameters, this method needs more first-principles calculations and is thus less efficient. Furthermore, the fitted result of the Heisenberg parameter $J_{ij}$ is vulnerable to the effects of other fourth-order interactions such as terms as $\left(S_{i}\cdot S_{j}\right)\left(S_{i}\cdot S_{k}\right)$ and $\left(S_{i}\cdot S_{j}\right)\left(S_{k}\cdot S_{l}\right)$. Therefore, the estimations of the Heisenberg parameters may not be very reliable if any of such fourth-order interactions are essential. This problem can be remedied by adding the related terms into the effective Hamiltonian model, while it is not easy to decide which terms to include in the model beforehand.

A possible way to get rid of the effects of other high-order terms is to perform artificial calculations where most of the magnetic ions are replaced by similar but nonmagnetic ions (e.g., substituting Fe^3+^ ions with nonmagnetic Al^3+^ ions) except for one or more ions to be studied [150]. For example, when calculating SIA, only one magnetic ion is not substituted, and by rotating this magnetic ion and calculating the total energy, the SIA can be studied. When studying two-body interactions between $S_{i}$ and $S_{j}$, only two magnetic ions (at site *i* and *j*) are not substituted, and by rotating the spins (or magnetic moments) of these two magnetic ions, the interactions between them can be studied. Such a technique of substituting atoms can be applied to energy mapping analysis based on either the four-state method or least-squares fitting. In this way, effects from interactions involving other sites are effectively avoided. Nevertheless, this substitution method can make the chemical environments of the remaining magnetic ions different from those in the system with no substitution. This may make the calculations of the interaction parameters untrustworthy.

#### 3.1.3. Methods Based on Expansions and Selecting Important Terms

The traditional energy-mapping analysis needs to construct an effective spin Hamiltonian first and then fit the undetermined parameters. However, it is not easy to give a perfect guess, especially when high-order interactions are essential. Such problems can be solved by considering almost all the possible terms utilizing some particular expansion with appropriate truncations. Usually, there are too many possible terms to be considered, so a direct fitting is impracticable; it requires at least as many first-principles calculations as the number of terms to determine, but leads to over-fitting problems due to too many parameters to determine. So, it is necessary to decide whether or not to include each term into the effective spin Hamiltonian on the basis of their contributions to the fitting performance.

In Ref. [145], SCE is adopted for the expansion of spin interactions of bcc and fcc Fe. After truncations based on the interacting distance and interaction orders, they considered 154 (179) possible different interaction terms in bcc (fcc) Fe. They randomly generated 3954 (835) different spin configurations in a 2 × 2 × 2 supercell for fitting. Their method of choosing terms is as follows: starting from the effective Hamiltonian model with only a constant term, try adding each possible term into the temporary model and accept the one providing the best fitting performance, in which way the terms are added to the model one by one. This method is the forward selection in variable selection problems, which is simple and straightforward, and it works well in most cases. However, this method may include unnecessary interactions to the effect Hamiltonian.

**Figure 5:**
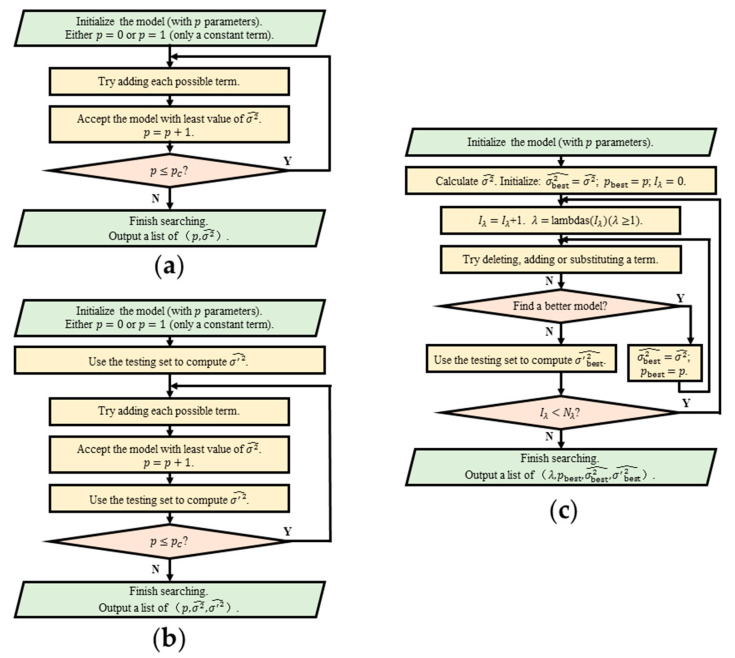
Flow charts of several methods of variable selection. In these flow charts, the fitting variance $\sigma^{2}$ estimated by the training set and the testing set are denoted as $\hat{\sigma^{2}}$ and $\hat{{\sigma^{\prime }}^{2}}$, respectively. (**a**) The traditional forward selection method as adopted in Ref. [145]. (**b**) The forward selection method using a testing set to check if over-fitting problems occur. (**c**) A simplified flow chart of the algorithm used in MLMCH [125], where some details are omitted. A testing set is used to check if over-fitting problems occur. The criterion for a better model is a smaller $\left(\hat{\sigma^{2}}\cdot \lambda^{p}\right)$ with λ≥1. There are $N_{\lambda }$ values of λ, which are set in advance, saved in the array “lambdas(1:$\;N_{\lambda }$)”, whose components are usually arranged in descending order of magnitude. For each value of λ, the best model is selected by using the criterion $\left(\hat{\sigma^{2}}\cdot \lambda^{p}\right)$.

In Ref. [125], a machine learning method for constructing Hamiltonian (MLMCH) is proposed, which is more efficient and more reliable than the traditional forward selection method. Firstly, a testing set is used to avoid over-fitting problems. Secondly, not only adding terms but also deleting and substituting terms are considered during the search for the appropriate model. Thus, if an added term is judged to be unnecessary, it can still be removed from the model afterward. A penalty factor $p^{\lambda }$ (λ≥1), where p is the number of parameters in the temporary model and λ is a given parameter, is used together with the loss function $\sigma^{2}$ (the fitting variance) to select models with fewer parameters. Several techniques are used to reduce the search space and enhance the search efficiency to select important terms out of tens of thousands of possible terms. The flow charts of this method of variable selection as well as the forward selection method are shown in Figure 5.

This method is advantageous in two ways: (a) Constructing the effective spin Hamiltonians is carried out comprehensively, which makes it less likely to miss some critical interaction terms; (b) this method is general, so it can be applied to most magnetic materials. The least-squares fitting needed in this approach can also provide the estimations for the uncertainties of the parameters. The flaw is that it needs lots of (typically hundreds of) first-principles calculations, which could be impracticable when a very large supercell is needed (especially when the material is metallic so that long-range interactions are essential). The way to generate spin configurations (typically randomly distributed among all possible directions, sometimes deviating only moderately from the ground state) may have some room for improvement.

### 3.2. Green’s Function Method Based on Magnetic-Force Linear Response Theory

In Green’s function method based on magnetic-force linear response theory [151,152,153,154,155,156,157,158,159], we need localized basis functions $\psi_{im\sigma }\left(r\right)$ (i,m,σ indicating the site, orbital, and spin indices, respectively) based on the tight-binding model. The localized basis functions can be provided by DFT codes together with Wannier90 [160,161] or codes based on localized orbitals. By defining

(24) $${ℍ}_{imjm^{\prime }\sigma \sigma^{\prime }}\left(R\right)=\langle \psi_{im\sigma }\left(r\right)|H|\psi_{im\sigma }\left(r+R\right)\rangle$$

(25) $$S_{imjm^{\prime }\sigma \sigma^{\prime }}\left(R\right)=\langle \psi_{im\sigma }\left(r\right)|\psi_{im\sigma }\left(r+R\right)\rangle$$

(26) $$ℍ\left(k\right)=\underset{R}{\sum }ℍ\left(R\right)e^{ik\cdot R}$$

(27) $$S\left(k\right)=\underset{R}{\sum }S\left(R\right)e^{ik\cdot R}$$

the Green’s function in reciprocal space and real space are defined as

(28) $$G\left(k,\varepsilon \right)={\left(\varepsilon S\left(k\right)-ℍ\left(k\right)\right)}^{-1}$$

and

(29) $$G\left(R,\varepsilon \right)=\int_{\mathrm{BZ}}^{}G\left(k,\varepsilon \right)e^{-ik\cdot R}dk$$

Based on the magnetic force theorem [162], the total energy variation due to a perturbation (which is the rotation of spins in this case) from the ground state equals the change of single-particle energies at the fixed ground-state potential:

(30) $$\delta E=\int_{-\infty }^{E_{F}}\varepsilon \delta n\left(\varepsilon \right)d\varepsilon =-\int_{-\infty }^{E_{F}}\delta N\left(\varepsilon \right)d\varepsilon$$

where

(31) $$n\left(\varepsilon \right)=-\frac{1}{\pi }Im\mathrm{Tr}\left(G\left(\varepsilon \right)\right)$$

and

(32) $$N\left(\varepsilon \right)=-\frac{1}{\pi }Im\mathrm{Tr}\left(\varepsilon -ℍ\right)$$

where traces are taken over orbitals. By defining

(33) $${\mathbb{P}}_{i}={ℍ}_{ii}\left(R=0\right)={𝕡}_{i}^{0}𝕝+{\vec{𝕡}}_{i}\cdot \vec{\sigma }$$

with its component

(34) $${\mathbb{P}}_{imm^{\prime }}=p_{imm^{\prime }}^{0}𝕝+p_{imm^{\prime }}{\vec{e}}_{imm^{\prime }}\cdot \vec{\sigma }$$

where $\vec{\sigma }$ is the vector composed of Pauli matrices. By defining

(35) $$G_{im,jm^{\prime }}=G_{im,jm^{\prime }}^{0}𝕝+{\vec{G}}_{im,jm^{\prime }}\cdot \vec{\sigma }$$

the energy variation due to the two-spin interaction between sites *i* and *j* is

(36) $$\delta E_{ij}=-\frac{2}{\pi }\int_{\infty }^{E_{F}}Im\mathrm{Tr}\left(\delta {ℍ}_{i}G\delta {ℍ}_{j}G\right)d\varepsilon$$

with $\delta {ℍ}_{i}=\delta \phi_{i}\times p_{i}$. After mathematical simplification, the expression of $\delta E_{ij}$ can be mapped to that given by the effective Hamiltonian model

(37) $$H_{s\mathrm{pin}}=\underset{i}{\sum }S_{i}^{T}A_{i}S_{i}+\underset{i,j>i}{\sum }\left[J_{ij}S_{i}\cdot S_{j}+D_{ij}\cdot \left(S_{i}\times S_{j}\right)+S_{i}^{T}K_{ij}S_{j}\right]+\underset{i,j>i}{\sum }K_{ij}{\left(S_{i}\cdot S_{j}\right)}^{2}$$

(including all the second-order terms and a biquadratic term) to obtain the expressions of the parameters:

(38) $$J_{ij}=Im\left(A_{ij}^{00}-A_{ij}^{xx}-A_{ij}^{yy}-A_{ij}^{zz}-2A_{ij}^{zz}S_{i}^{\mathrm{ref}}\cdot S_{j}^{\mathrm{ref}}\right)$$

(39) $$K_{ij}^{uv}=Im\left(A_{ij}^{uv}+A_{ij}^{vu}\right)$$

(40) $$D_{ij}^{u}=Re\left(A_{ij}^{0u}-A_{ij}^{u0}\right)$$

(41) $$B_{ij}=Im\left(A_{ij}^{zz}\right)$$

where

(42) $$A_{ij}^{uv}=\frac{1}{\pi }\int_{-\infty }^{E_{F}}\mathrm{Tr}\left\{{𝕡}_{i}^{z}G_{ij}^{u}{𝕡}_{j}^{z}G_{ji}^{v}\right\}d\varepsilon$$

with u,v∈{0,x,y,z} (the trace is also taken over orbitals), and $S_{i}^{\mathrm{ref}}$ indicates the unperturbed vector $S_{i}$. An *xyz* average strategy can be adopted so that some components inaccessible from one first-principles calculation can be obtained [157].

The main advantages of this method are that it only requires one or three DFT calculations to obtain all the parameters of second-order terms and biquadratic terms between different atoms, using only a small supercell (with a dense enough *k*-point sampling) to obtain interaction parameters between spins far away from each other. Therefore, it saves the computational cost compared to the energy-mapping analysis, especially when long-range interactions are essential. It also avoids the difficulties of reaching self-consistent-field convergence in DFT calculations for high-energy configurations, which may occur in the energy-mapping analysis. This method is good at describing states near the ground state but may not be so good at describing high-energy states. A limitation is that this method cannot obtain SIA parameters, and its calculations for biquadratic parameters are not very trustworthy [157]. The calculated Heisenberg parameter $J_{ij}$ is mixed with the contributions of other fourth-order interactions such as terms like $\left(S_{i}\cdot S_{j}\right)\left(S_{i}\cdot S_{k}\right)$ and $\left(S_{i}\cdot S_{j}\right)\left(S_{k}\cdot S_{l}\right)$. Therefore, the results may be unreliable if any of such fourth-order interactions are essential. Another little flaw is the noise of a typical order of magnitude of a few μeV introduced by the process of obtaining the Wannier orbitals [157]. In addition, the uncertainties of the parameters cannot be obtained by this method.

A recent study [140] considered the rotations of more than two spins as the perturbation and mapped the δE to the corresponding quantity given by an effective Hamiltonian with more types of interactions, including terms proportional to $S_{i}\cdot \left(S_{j}\times S_{k}\right)$, $\left(S_{i}\cdot S_{j}\right)\left(S_{k}\cdot S_{l}\right)$ and $\left(S_{i}\times S_{j}\right)\left(S_{k}\cdot S_{l}\right)$. This enables one to obtain the expressions needed for calculating the associated parameters. The derivations and forms of the expressions are much more complicated compared with those from the second-order interactions discussed above. This generalization of the traditional approach for calculating second-order interaction parameters remedied the problem of ignoring the effects of other high-order interactions to some extent. However, it is still a challenging task to get rid of the effects of high-order interactions on calculating the Heisenberg parameters (and other second-order parameters). The noise introduced by Wannier orbitals, the inability to determine SIA and the uncertainties of the resulting parameters are still the drawbacks of this approach. In addition, this method cannot give the derivatives of exchange interactions with respect to the atomic displacements, in contrast to the four-state method discussed above.

### 3.3. More Discussions on Calculating Spin Interaction Parameters

We should notice that, for all the methods discussed above, a rigid spin rotation approximation is used. The latter is equivalent to the supposition that the magnitudes of the spins should be constant in different configurations. However, this is not always true. For example, the magnitudes of the spins may be a little different in FM and AFM states. In Ref. [128], the energy-mapping analysis based on direct least-squares fitting (with configurations generated with different θ values) is adopted to study the spin interactions of MnGe and FeGe, to find that the agreement between the calculations and the model is enhanced by allowing the magnitudes of the spins to depend on the parameter θ (which decides the configurations) instead of using the fixed magnitudes (see Supplementary Materials of Ref. [128]). It is possible to obtain the relationship between the magnitudes of the spins and the parameter θ with an appropriate fitting or interpolation so that for a configuration with a new value of θ, the magnitudes of spins and the total energy can be predicted. Nevertheless, for a general spin configuration that cannot be described by a single θ, the prediction for the magnitudes of the spins can be very difficult. This is why one commonly employs the effective spin Hamiltonian by assuming that the magnitudes of the spins are constant.

Another perspective for the rigid spin rotation approximation, as implied in Ref. [143], is that even if the magnitudes of the spins are highly relevant to the configurations, the total energy can be fitted by using the directions of spins, instead of the spin vectors themselves, as the independent variables (which is mathematically equivalent to supposing the magnitudes of the spins to be constant) and considering an appropriate expansion of these variables (spin directions). For example, supposing SOC can be ignored (supposing SOC is switched off during first-principles calculations), the Heisenberg term $J_{ij}S_{i}\cdot S_{j}$ can be expressed as $J_{ij}S_{i}\cdot S_{j}=J_{ij}S_{i}e_{i}\cdot S_{j}e_{j}$; the magnitudes of the spins $S_{i}$ and $S_{j}$ depend on the angles between these two spins or neighboring spin directions (e.g., $e_{k}$). Therefore,

(43) $$J_{ij}S_{i}\cdot S_{j}={\tilde{J}}_{0,\;ij}e_{i}\cdot e_{j}+C_{1}{\left(e_{i}\cdot e_{j}\right)}^{2}+C_{2}\left(e_{i}\cdot e_{k}\right)\left(e_{i}\cdot e_{j}\right)+C_{3}\left(e_{j}\cdot e_{k\prime }\right)\left(e_{i}\cdot e_{j}\right)+\cdots .$$

That is to say, the relevance of the magnitudes of spins to the configurations can be transferred to higher-order interactions when supposing the magnitudes of spins to be constant. These “artificial” higher-order terms, only emerging for compensating for such configuration dependency, are not very physical but can somewhat improve the fitting performances (when such dependency is prominent).

All the methods discussed above assumes, besides the rigid spin rotation approximation, that magnetic moments are localized on the atoms. In addition, we note that the DFT calculation results depend on the chosen exchange-correlation functional and the value of DFT+U parameters [157].

In the above discussions, we have supposed the periodic boundary conditions, for we mainly focus on the studies of crystals. When dealing with clusters (e.g., single-molecule magnets), we can still arrange a cluster in a crystal (using periodic boundary conditions) [159] with enough vacuum space to prevent the interactions between two clusters belonging to different periodic cells. If no periodic boundary conditions exist, the energy-mapping analysis can still work, while Green’s function method based on magnetic-force linear response theory will fail because the reciprocal space is not defined. For the cases without periodic boundary conditions, theoretical chemists have developed several other approaches (such as wave-function based quantum chemical approaches) for studying magnetic interactions [69,146,147,163,164,165], detailed discussions of which are beyond the scope of this review.

The spin interaction parameters can also be obtained from comparing the experimental results of observable quantities such as transition temperatures, magnetization [166], specific heat [166], magnetic susceptibility [166,167], and magnon spectrum (given by inelastic neutron scattering measurements) [110,124,168,169,170,171] with the corresponding predictions given by the effective Hamiltonian model, which is similar to the idea of energy-mapping analysis based on least squares fitting. While the transition temperatures can only be used to roughly estimate the major interaction (typically the Heisenberg interaction between nearest pairs), the magnon spectrum can provide more detailed information and thus widely adopted for obtaining interaction parameters. These experimental results can also be used for checking the reliability of effective spin Hamiltonian models and the corresponding parameters obtained from first-principles calculations [172].

## 4. Conclusions

In this review, we summarized different types of spin interactions that an effective spin Hamiltonian may include. Recent studies have shown the importance of several kinds of high-order terms in some magnetic systems, especially biquadratic terms, four-spin ring interactions, topological chiral interactions, and chiral biquadratic interactions. In addition, we discussed in some detail the advantages and disadvantages of various methods of computing interaction parameters of the effective spin Hamiltonians. The energy-mapping analysis is easier to use, and it is less vulnerable to the effects of higher-order interactions (if carefully treated). Compared with the energy-mapping analysis, Green’s function method requires less first-principle calculations and a relatively small supercell. The energy-mapping analysis usually gives a relatively good description of many kinds of states with diverse energies, while Green’s function method provides a more accurate description of states close to the ground state (or the reference state). Both methods usually provide similar results and are both widely adopted in the studies of magnetic materials. We expect that first-principles based effective spin Hamiltonian will continue to play a key role in the investigation of novel magnetic states (e.g., quantum spin liquid and magnetic skyrmions).
